# Resection of Parosteal Osteosarcoma of the Distal Part of the Femur: An Original Reconstruction Technique with Cement and Plate

**DOI:** 10.1155/2008/763056

**Published:** 2008-10-20

**Authors:** F. Pezzillo, G. Maccauro, T. Nizegorodcew, B. Rossi, G. Gosheger

**Affiliations:** ^1^Orthopaedic Department, Catholic University, UCSC Pol. Gemelli, 8-00168 Rome, Italy; ^2^Department of Orthopaedics, Münster University, 48147 Münster, Germany

## Abstract

Parosteal osteosarcoma is a low-grade malignant bone tumor arising from the distal femur and tibia. Wide resection of a parosteal osteosarcoma usually prevents local recurrence. In literature, hemicortical resections of low-grade malignant bone tumors and allograft reconstruction are described. We describe a new method of resection and reconstruction of parosteal osteosarcoma located in the popliteal paraosseous space of the distal part of the femur using cement and plate (LISS-SYNTHES) through dual medial and lateral incisions. The patient did not present infections and fractures and the functional results were good. After one year, no metastases developed and there were no local recurrences.

## 1. INTRODUCTION

Parosteal osteosarcoma is a rare form of low-grade malignant osteosarcoma which usually arises on the surface of the metaphysis of long bones, representing 1–6% of all osteosarcoma [1–3]. It affects females more often than males and the peak of incidence is in the third and fourth decades [4]. This tumor is usually well differentiated low-grade lesions with a low propensity to metastasize, and the prognosis is better compared with conventional osteosarcoma [5]. Parosteal osteosarcoma tends to be large at presentation and its predilection for posterior aspect of distal femur has made achieving wide surgical margins difficult as only loose fibro-fatty tissue separates the tumor from adjacent neurovascular structures [6]. When the tumor involves the medullar canal, it usually does not occupy more than 25% of the canal's diameter [7]. Resection with a wide operative margin is the most appropriate method of treatment [5]. Treatment with wide resection and reconstruction with a prosthesis has been advocated [8], but when the parosteal osteosarcoma is in the distal part of the femur this requires extensive dissection of the surrounding soft tissues and the neurovascular bundle, removal of the articular surface of the distal part of the femur, and replacement of the entire distal femur and proximal tibia. In the literature, a technique of hemicortical resection and allograft reconstruction is described [9]. We describe an original reconstruction technique with cement and plate (LISS-SYNTHES).

## 2. CASE REPORT

**Figure 1:**
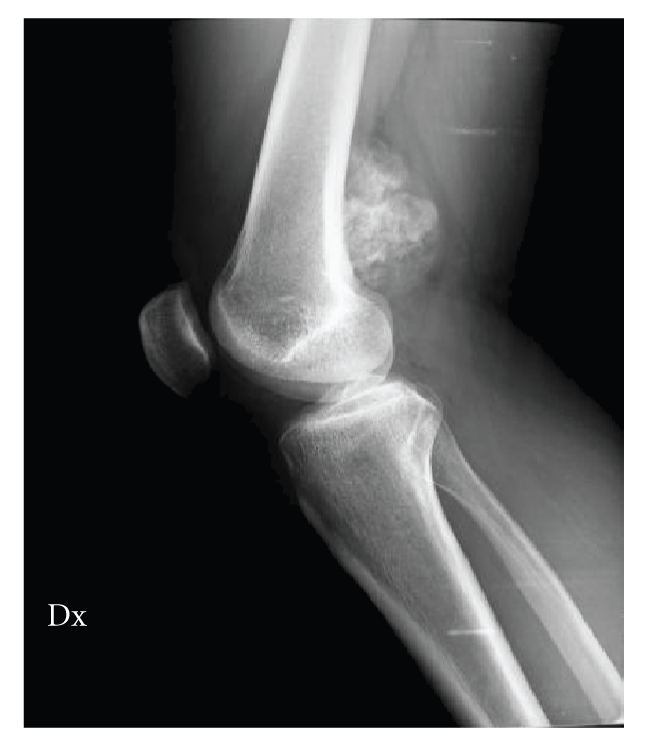
Preoperative radiography showed a relatively dense and well-demarcated ossified mass at a juxtacortical lesion of the distal femur.

**Figure 2:**
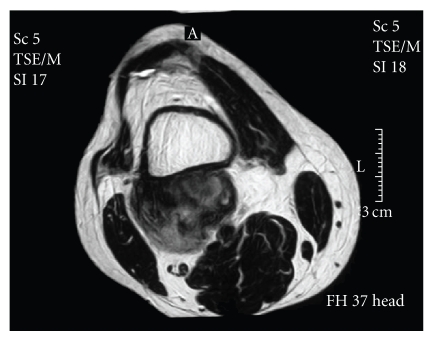
Preoperative MRI showed a relatively dense and well-demarcated ossified mass at a juxtacortical lesion of the distal femur without medullar involvement.

**Figure 3:**
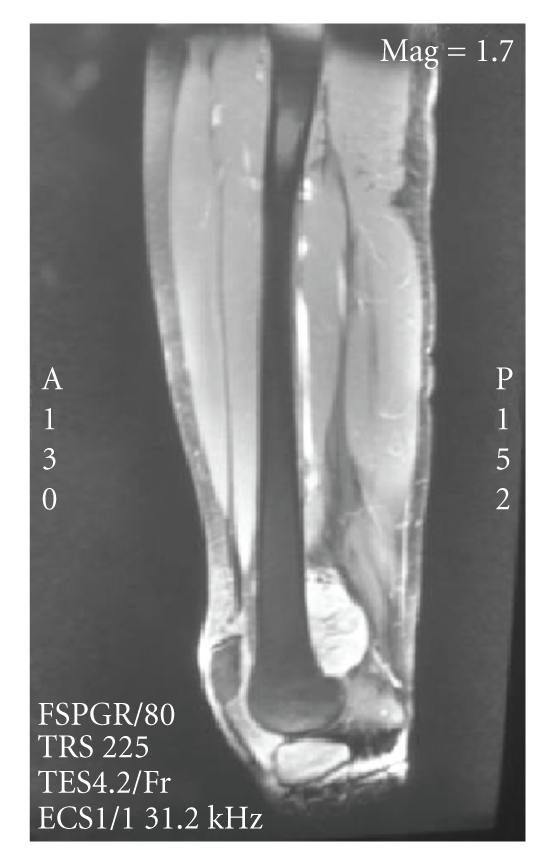
Preoperative MRI showed a relatively dense and well-demarcated ossified mass at a juxtacortical lesion of the distal femur without medullar involvement.

**Figure 4:**
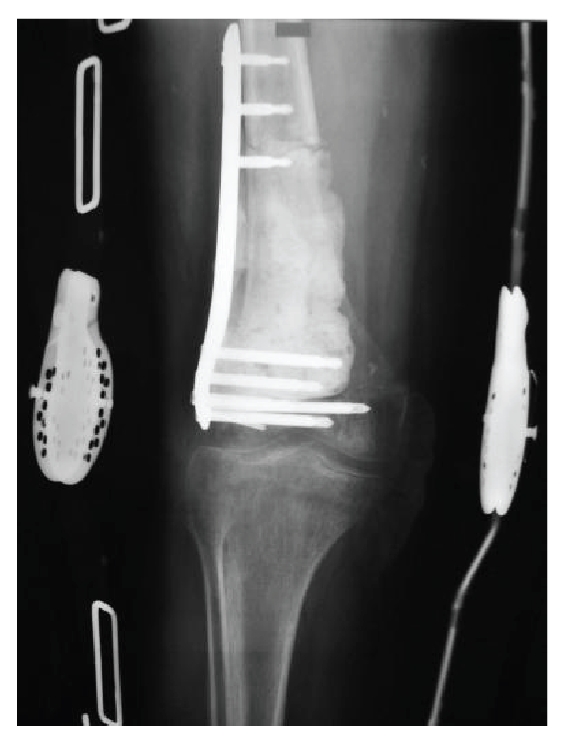
Cement, plate (LISS-SYNTHES), and screws were used for the reconstruction of the distal femoral defect.

**Figure 5:**
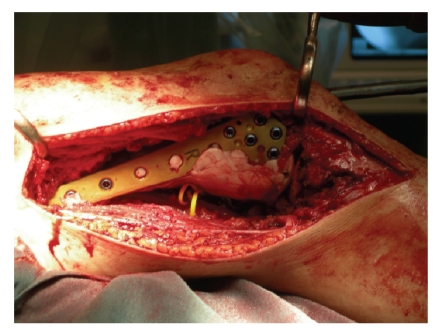
The plate, cement, and screws used to reconstruct the bone defect.

**Figure 6:**
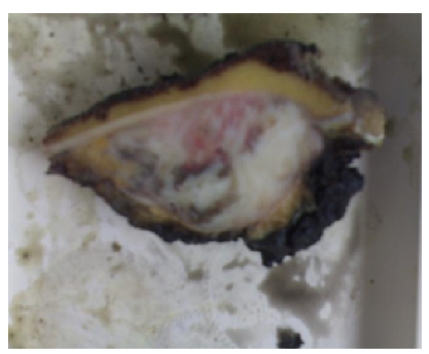
The tumor had minimal focal spread into the medullar canal (less than 10% of total surface) and did not show invasion into the overlying muscles.

A 34 year-old male arrived to our department after a 6 months history of sever pain and swelling located on the posterior cortex of the distal right femur. Laboratory data showed a slightly increased value of alkaline phosphatase. Local preoperative staging of the tumor was done using plain radiography and MRI with T1 and T2 weighted sequences and contrast-enhanced series with gadolinium. Preoperative radiography (Figure 1) and MRI showed a relatively dense, well-demarcated ossified mass at a juxtacortical lesion of the distal femur, which indicated a low-grade bone tumor, without medullar involvement (Figures 2 and 3). Total body CT and MRI did not show skip or lung metastases. The nature of the tumor was established by histological examination of the lesion at the time of the resection. The site of resection and osteotomy was determined on the basis of preoperative MRI and radiography. Surgical technique was according to Lewis et al. [10]. The patient was positioned supine. The knee was flexed to 65 degrees and the foot was fixed to the operating table. A longitudinal incision was made from the medial midportion of the thigh to five centimeters distal to the knee. The femoral vein and artery were identified in the popliteal space. The medial part of distal femur was exposed. A similar dissection was made on the lateral side of the distal femur and the lateral part of distal femur was exposed. The popliteal space was identified through the dissection between the long and the short heads of the biceps femora muscle. On the basis of the preoperative MRI and radiography used to study the extension of the tumor we made the longitudinal medial and lateral osteotomy and the proximal transverse osteotomy. The resected tumor was removed and the pathologist examined the specimen to determine if the margin was adequate. Cement, plate (LISS-SYNTHES), and screws were used for the reconstruction of the distal femoral defect (Figure 4). Cement Methylmethacrylate with tobramycin was obtained in the form of powder and a liquid monomer (Surgical Simplex P, Howmedica Antibiotic). The preparation included 40 g of powder and 20 mL of monomer. The cement mixtures were made by mixing the cement powder and the liquid monomer under vacuum using dedicated instrumentation (Simplex Enhancement Mixer, Howmedica). After mixing for 100 seconds, the vacuum was removed and the cement was used. We applied the plate after we fixed it with 2 proximal and 3 distal screws in the cortical bone. Then we applied the cement to reconstruct the bone defect fixing the plate with other screws in the cement (Figure 5). After 3 days, the patient was allowed to walk with toe-touch weight-bearing using crutches and to move the knee. Progressive weight-bearing is allowed and the patient was fully weight-bearing after 1 month. After the resection, specimen was examined to determine the histological grade, the intramedullar involvement, and the operative margin. The tumor had minimal focal spread into the medullar canal (Figure 6) (less than 10% of total surface) and did not show invasion into the overlying muscles. Wide margin resection by Enneking classification [9] was observed. No postoperative wound complications and infection occurred. After 1 year, the patient does not present local recurrences or distant metastases. The range of motion of the knee is 0 to 110 degrees and the patient returned to his preoperative life.

## 3. DISCUSSION

**Figure 7:**
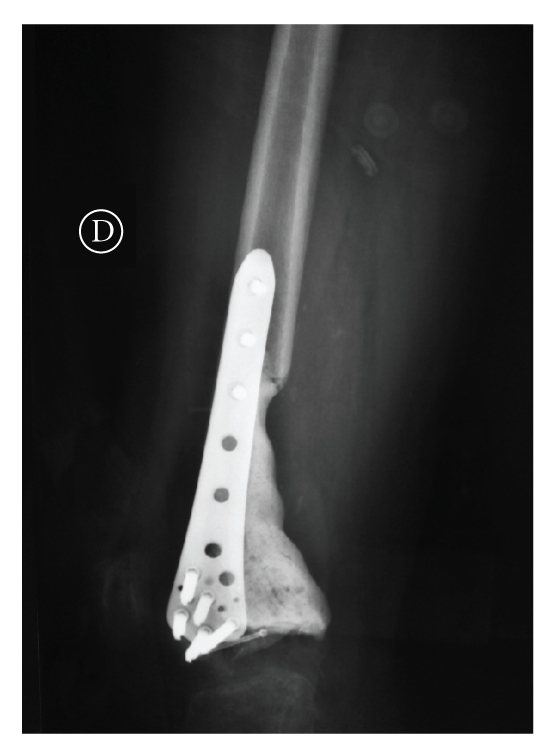
Lateral radiography of the reconstructive technique at the one-year follow-up.

Parosteal osteosarcoma is an uncommon lesion, low-grade and well-differentiated tumor with rare or limited involvement of the medullar canal [5]. Many procedures were described in the literature for the treatment of parosteal osteosarcoma [4–6, 10]. Inadequate resection can cause local recurrence and distant metastases [6]. Marginal but histological negative surgical margin seems adequate for local control of parosteal osteosarcoma and to prevent distant metastases [10, 11]. Tumor extension into the medullar canal has been reported; it may necessitate the resection of the entire segment of bone and the reconstruction with prosthesis. The analysis of the patients with parosteal osteosarcoma treated at the Mayo Clinic demonstrated no association between local recurrence and medullar involvement [7]; moreover, other studies showed no association between local recurrence and medullar involvement [5, 10, 11]. In the cases located in the posterior part of distal femur, which is the most frequent location of parosteal osteosarcoma, we perform a marginal excision (hemicortical resection) if the articular surface of the distal femur can be saved. Medullar involvement may occur, but we considered that it is not a contraindication for the hemicortical resection. In this case, the minimal focal spread into the medullar canal did not prevent to obtain a wide surgical margin. This seems possible because invasions usually involve less than 25% of the medullar canal [7, 12]. Marginal excision usually results in hemicortical defect of the bone usually reconstructed with allograft [10]. In the literature, six cases of fractures of the remaining hemi cortex and two cases of minor subcortical resorption of the graft in the report of 22 cases of hemicortical allograft reconstruction after resection of low-grade malignant bone tumors are described. Reconstruction with allograft was not judges indicated when large resection was done, as in our case [12]. We used cement and plate (LISS-SINTHES) to reconstruct the bone defect, and we think that this is a new valid and alternative technique when it is not possible to use allograft and to prevent allograft complications such as fractures and bone resorption. Moreover, bone cement allows early postoperative weight bearing, thanks to the mechanical properties of PMMA. In the literature, cement is currently used for filling bone defect after curettage in bone oncology [13, 14] and more recently is described the use of the cement in other district such as tibia [15] to reconstruct the bone defects. It is well known the possibility of bone cement to give mechanical resistance to involved segment [16, 17] and to evaluate early tumor recurrence [17]; a longer follow-up in the time will show us the mechanical resistance of the cement in a large area exposed to the load and the wear in the time. We think that alternative indications for this technique could be metastatic tumors as opposed to prostheses or cadaveric allograft for the reconstruction of bone defect. In our technique, the femoral articular surface is saved, the joint remains stable, and the knee function is good 1 year after the surgery (Figure 7). In presence of complication, bone resection and reconstruction with tumoral prostheses should be considered.
